# Interventions to mitigate the risks of COVID-19 for people experiencing homelessness and their effectiveness: a systematic review

**DOI:** 10.3389/fpubh.2023.1286730

**Published:** 2024-01-04

**Authors:** Obianuju Ogbonna, Francesca Bull, Bethany Spinks, Denitza Williams, Ruth Lewis, Adrian Edwards

**Affiliations:** ^1^Health and Care Research Evidence Centre, Division of Population Medicine, School of Medicine, Cardiff University, Cardiff, United Kingdom; ^2^Division of Population Medicine, School of Medicine, Cardiff University, Cardiff, United Kingdom; ^3^North Wales Centre for Primary Care Research, School of Medical and Health Sciences, Bangor University, Bangor, United Kingdom

**Keywords:** homelessness, COVID-19 pandemic, systematic review, public health impacts, intervention

## Abstract

**Objectives:**

People experiencing homelessness also experience poorer clinical outcomes of COVID-19. Various interventions were implemented for people experiencing homelessness in 2020–2022 in different countries in response to varied national guidance to limit the impact of COVID-19. It is important to understand what was done and the effectiveness of such interventions. This systematic review aims to describe interventions to mitigate the risks of COVID-19 in people experiencing homelessness and their effectiveness.

**Methods:**

A protocol was developed and registered in PROSPERO. Nine databases were searched for studies on interventions to mitigate the impact of COVID-19 on people experiencing homelessness. Included studies were summarised with narrative synthesis.

**Results:**

From 8,233 references retrieved from the database searches and handsearching, 15 were included. There was a variety of interventions, including early identification of potential COVID-19 infections, provision of isolation space, healthcare support, and urgent provision of housing regardless of COVID-19 infection.

**Conclusion:**

The strategies identified were generally found to be effective, feasible, and transferable. This review must be interpreted with caution due to the low volume of eligible studies and the low quality of the evidence available.

## 1 Introduction

When the COVID-19 pandemic began, many experts raised concerns over the clinical vulnerability of people experiencing homelessness to COVID-19 due to the higher prevalence of long-term conditions, infection, or mental illness (1–6). Social and environmental factors were also significant determinants, over and above the main risk factors for the whole population, including demographic indicators (population density, ageing population, *per capita* income, etc.), environmental variables (temperature, humidity, etc.), healthcare, and infrastructure facilities (7, 8). Some people experiencing homelessness live in congregate settings such as shelters, where large numbers live in enclosed spaces with a higher risk of infection spread (9). Rough sleepers, an extreme form of homelessness, often have complex health needs and are at high risk of impacts from extreme temperatures and malnutrition (10, 11).

Variations in available accommodation and healthcare are seen between homeless populations globally. The US predominantly uses shelters for people experiencing homelessness (12), whereas the UK has shifted to using more hostel-type accommodations (13). Nonetheless, interventions implemented may have features in common as they often include congregate living and can be adapted and applied to people experiencing homelessness regardless of location (14).

National guidance for the general population could not always be acted upon by people experiencing homelessness, such as policies in the UK to stay at home, social distancing, and frequent handwashing (15). Actions specific to people experiencing homelessness were required and put in place to mitigate COVID-19 risks, ranging from small-scale interventions to national policy (16). For example, the UK aimed to house all rough sleepers in accommodation to mitigate the risks of infection and help their ability to isolate. England adopted the initiative known as *Everyone In,* and the Welsh Government funded a similar approach (17, 18).

Implementing interventions in this population can be difficult, and most research on interventions for disease outbreaks in homeless populations (prior to COVID-19) lacked formal evaluation of the implementation and effectiveness of interventions (19). An interim report examined and compared the UK devolved nations’ responses to homelessness during COVID-19 between March and December 2020, focussing on policies and funding (20). However, this report does not cover smaller initiatives and does not evaluate the effectiveness of interventions relating to COVID-19 clinical outcomes (e.g., prevalence, hospitalisation, mortality, long COVID, and mental health impact). These are important to consider as interventions could potentially cause more harm than good, and be costly, especially considering how many unknowns there were at the onset of the COVID-19 pandemic.

There is a wide variety and scale of potential interventions internationally. This systematic review aims to describe interventions to mitigate the risks of COVID-19 in people experiencing homelessness and their effectiveness. This is especially pertinent considering the potential need for managing future waves of the COVID-19 pandemic or other infections, to address health inequalities and identify further research that is necessary in such events in the future.

## 2 Method

A protocol for this systematic review was developed and registered in PROSPERO (PROSPERO registration 2022 CRD42022304941). This review was conducted in accordance with good practice guidelines (21), and reporting was guided by the standards of the PRISMA statement (22).

### 2.1 Selection criteria

The selection criteria for this review were developed with guidance from stakeholders with expert knowledge of public health and homelessness (Table 1).

**Table 1:** Eligibility criteria used for selecting studies in the review.

	Inclusion criteria	Exclusion criteria
Population	People aged 16 and over experiencing homelessness during COVID-19 pandemic. Using the ETHOS definition of homelessness (23) Country of origin: upper and middle income countries.	Studies that do not include research on people experiencing homelessness. Populations in low-income countries People under the age of 16.
Intervention	Single or multi-component intervention(s) that had the aim of reducing a risk or complication of COVID-19 in the people experiencing homelessness (e.g., testing in a homeless shelter and temporary housing initiatives). Interventions targeted at an individual, centre or population level.	Studies that do not describe an intervention with an aim relating to the COVID-19 pandemic. E.g., an intervention to reduce smoking rates. Interventions to prevent homelessness, e.g., a policy to prevent evictions by landlords.
Comparisons	Comparison of outcomes to a historical control from before to during the pandemic or to another similar population without intervention	Opinion pieces; Studies with no comparison. Systematic reviews (reference lists checked)
Outcome measures	Studies reporting clinical outcomes related to COVID-19: Rates of COVID-19 transmission Hospitalisation and mortality rates relating to COVID-19 infection Healthcare safety incidents Mental health impact Long COVID rates	Clinical outcomes of COVID-19 not measured (or described)
Language	English	Not published in English
Publication date, type	During / since start of pandemic, published and preprint	

### 2.2 Search strategy

The search strategy and choice of databases searched were assisted by a subject librarian. Key concepts of COVID-19 and homelessness were used, aiming for a high recall of relevant articles. The COVID-19 search string was derived from international evidence synthesis resources (12, 17). The search string for homelessness was developed from published systematic review searches (12, 19) and in accordance with the ETHOS definition (23).

The search strategy was developed and run on MEDLINE (OVID) and then adapted for use on the following databases: Embase, CINAHL, Cochrane Library, ASSIA, Web of Science, L*VE Evidence, Social Policy and Practice, and Scopus in November 2022. The search strategy and results from the MEDLINE search are presented in Appendix. Studies still in the stage of pre-print were covered with the Embase and L*VE Evidence databases. Follow-up sources such as submissions from stakeholders and reference list checking were also used.

### 2.3 Study screening and selection

The results from each database were exported onto the reference management software EndNote (24). Duplicates, studies published before 2020, and those not in English were excluded. Deduplication was carried out via EndNote. The remaining references were screened for eligibility using the criteria in Table 1.

Titles and abstracts were screened first by FB or UO, and 10% of the results were randomly selected to also be screened by another team member (BS) to assess consistency. Disagreements in selection were minimal, and so were discussed and resolved between the two reviewers without the involvement of a third. The full texts of potentially relevant studies were then screened by a single reviewer (FB or UO).

Based on background reading, it was predicted that there would be a low number of robust, high-quality eligible studies for this systematic review. Therefore, a hierarchy of evidence was used to prioritise higher-level study designs and not exclude lower-level evidence sources if eligible (25).

### 2.4 Data extraction

A form on Microsoft Excel was tested and used to extract relevant study details: title and authors, setting, population, study design and methodology, study period, intervention and comparison, outcomes measured, main conclusions, and limitations as reported in the study (abridged version in Table 2).

**Table 2:** Summary of included studies.

Study title, authors, year, country	Design study period	Population/setting, sample size (*n*)	Intervention	Comparison I outcomes (O)	Methodology	Main conclusions and key results	Overall assessment of quality limitations as reported and methodological appraisal
Early identification of a COVID-19 outbreak detected by wastewater surveillance at a large homeless shelter in Toronto, Ontario (26) Akingbola et al., 2022, Canada	Quasi-experimental January 2021	Men’s homeless shelter *N* = 169	Wastewater surveillance for COVID-19	C:with other homeless shelters/previous wastewater surveillance but not clear O:detection of COVID-19 from wastewater surveillance	Wastewater samples taken over 1 h retrieved twice weekly from the site. The surveillance team were made aware of any COVID-19 symptoms/cases before commencement of surveillance	Wastewater surveillance enabled prompt dissemination of COVID-19 testing in asymptomatic patients, therefore facilitating effective outbreak management	Low no comparison, no follow-up, no mention of inter-rater reliability, n relatively small of only 169
Implementation of Rapid and Frequent SARS-CoV2 Antigen Testing and Response in Congregate Homeless Shelters (27) Aranda-Diaz et al. 2021, United States	Prevalence study January–February 2021	Homeless shelters *n* = 828	Testing strategy and isolation provision	C: between stages of intervention, demographics O: detecting COVID-19 infection, isolation, identify outbreaks	Programme of regular COVID-19 antigen testing in 10 congregate living shelters. Implemented for residents and staff. Positive individuals were referred to isolation and contact-tracing done. Used RE-AIM framework to guide implementation and evaluation.	Testing and isolation strategy was able to be implemented effectively, detect COVID-19 infections, isolate individuals and identify outbreaks. 47.5% eligible residents participated in testing at least once. Identified 10 positive cases, 8 successfully isolated.	Very low Low participation rate, low adherence to twice-weekly testing, does not have control, short study period. Some limitations in transferability to UK given setting, may be applied to other congregate settings.
Clinical Outcomes, Costs, and Cost-effectiveness of Strategies for People Experiencing Sheltered Homelessness During the COVID-19 Pandemic (28) Baggett et al., 2020, United States	Modelling study April–August 2020	Homeless shelters *n* = 2,258	Symptom screening, regular testing, alternative care sites (ACSs), temporary housing	C: No intervention O: cumulative infections and hospital days, costs to healthcare sector, cost effectiveness	Decision analytic model using a simulated cohort residing in homeless shelters, based on literature and national databases. Looked at disease progression, transmission, and outcomes.	From the model: Daily symptom screening with alternative care sites for pending or confirmed cases of COVID-19 was associated with fewer severe COVID-19 infections (37%) and decreased healthcare costs (46%) in the homeless population. Fortnightly PCR testing and temporary housing most effective (81% fewer infections) but much higher costs (542%) % increase/decrease compared with no intervention	Low Findings were specific to individual adults, homeless families and rough sleepers excluded. Assumed homogenous mixing in shelters which may alter infections projected in model. Did not factor in higher rates of comorbidities in homeless population. Focussed on one location with different cost of living to other areas. Limited transferability given based on US setting and costs but demonstrates times where prevention cheaper than healthcare treatment costs.
Comparison of infection control strategies to reduce COVID-19 outbreaks in homeless shelters in the United States: a simulation study (29) Chapman et al., 2021, United States	Modelling study March–April 2020	Homeless shelters *n* = not stated	Daily symptom screening, PCR testing, universal masking, relocation of possibly infected individuals, staff testing	C: no intervention O: probability of averting an outbreak	Developed individual-level microsimulation model of COVID-19 transmission in homeless shelters and calibrated to data from PCR surveys across 5 shelters and 3 cities. Assessed risk (low, medium, high) of shelter characteristics, e.g., distancing, volume density.	Combination of strategies (symptom screening, regular testing, relocation, mask wearing) most effective. High risk settings (i.e., high density, high rates in background population) showed little improvement with any strategy. Daily symptom screening ineffective at all levels of transmission (probability of preventing outbreak = 0.04). Combining this with relocating of individuals with high-risk clinical symptoms combined did not improve outcomes. PCR testing 2x weekly for all individuals and universal mask wearing improved probability of averting outbreak better than symptom screening.	Low Limited data availability meant study only calibrated model to small number of shelter outbreaks, cross-sectional nature, assume equal transmissibility in model, simplifying assumptions, short study period. Some transferability to UK as the interventions studied are relevant to UK populations.
Assessment of contact tracing for COVID-19 among people experiencing homelessness, Salt Lake County Health Department, March–May 2020 (30) Fields et al., 2021, United States	Cross-sectional survey (prevalence) March–May 2020	Homeless shelters (majority), *n* = 169	Contact tracing	C: general population O: follow-up, number of contacts identified	Homeless people with laboratory confirmed positive COVID-19 cases documented in surveillance system included in analysis. A general population comparison group was systematically selected from all confirmed cases identified during same period. Person-based contact tracing through interviews asking about contacts, living place, businesses	Challenges in identifying, locating, and reaching cases among homeless population and their contacts. 55% of homeless with positive COVID-19 cases were interviewed (73 uncontactable, 3 refusals) compared to 100% general population. 81% of homeless reported no contacts. Homeless were more likely to be lost to follow-up compared to general population (14.2% vs. 0%, *p* < 0.0001), contacts of homeless were more often unreachable (13% vs. 7% *p* < 0.0001). COVID-19 testing completed for 62% (31) of the homeless contacts (42.5%, 322 general population). 16% of homeless contacts compared to 22% of gen pop contacts tested positive *p* = 0.3	Moderate Contact tracing findings from this district may not be transferrable to other areas, also contact tracing done early in pandemic. Does not actually identify reasons for homeless having fewer contacts, more difficult follow up. Low number of women in sample. May be cases of COVID-19 undocumented in homeless. Some transferability given transient nature of most homeless population regardless of country of origin.
Assessment of a Hotel-Based COVID-19 Isolation and Quarantine Strategy for Persons Experiencing Homelessness (31) Fuchs et al., 2021, United States	Prevalence study March–May 2020	Multiple homeless categories, *n* = 1,009	Hotel-based COVID-19 isolation with some healthcare	C: Between Subgroups of homeless population, demographics O: programme retention/premature discontinuation of quarantine	Hotel-based care system: individuals unable to safely isolate at home (with mild–moderate COVID-19 infection, pending test, close contact), were referred from other settings (hospitals, outpatients, public health surveillance) Physician-supervised team of nurses and others offered care and monitoring.	Hotel-based isolation strategy that delivered integrated health support for homeless people was implemented safely outside of hospital, adherence was fairly high (81%), although significant association of premature discontinuation with unsheltered (aOR 4.5, 95% CI, 2.3–8.6). Other risk factors were: being a close contact (aOR 2.6), age < 40 (2.5), female (1.8), black ethnicity (1.7). Used sensitivity analysis and regression models.	Low Missing data on homelessness, results on hospital stay are limited due to times of pandemic, may not be generalisable to all settings due to reliance on other workers outside of public-health. Design listed as retrospective cohort, but no non-exposure group. Some transferability to UK, evidence of successful implementation of hotel isolation which incorporates care, evidence of issues with housing rough sleepers.
Implementation of a Recuperation Unit and Hospitalisation Rates among People Experiencing Homelessness with COVID-19 (32) Gai et al., 2021, United States	Pre/post-intervention March–June 2020	Unspecified homelessness, *n* = 226	COVID-19 recuperation unit (CRU)	C: pre-intervention O: hospitalisation rates	Analysis of COVID-19 hospitalisation census from a single hospital. COVID-19 recuperation unit (CRU) opened midway through study period, provided isolation and quarantine for homeless and treatment for substance use.	An alternative care site for homeless with COVID-19 infection was associated with a reduction in hospitalisations in the homeless population. There was a 28% reduction in hospitalisations pre/post-intervention (risk ratio 0.72, 95% CI, 0.63–0.82)	Low May have missed hospitalisations elsewhere as only one hospital. Some transferability to UK, implementation of hotel isolation, however, does not specify homeless type.
Comparison of COVID-19 mitigation and decompression strategies among homeless shelters: a prospective cohort study (33) Hsu et al., 2021, United States	Prospective cohort study March–May 2020	Homeless shelters, *n* = 381	Depopulation strategies: provision of lodging in temporary tents in car park, gym, and hotel spaces	C: between interventions O: rates of COVID-19 infection	Study looks at residents in two homeless shelters which adopted different strategies to reduce density of shelters. Guests from one shelter were distributed to recreational centre space, hotel, while the other in temporary tents in car park. COVID-19 testing and pre + post-test survey	Depopulation strategies to multiple different locations of stable accommodation was better at preventing COVID-19 infection compared with outdoor tent set-up. Tent intervention participants had 6.21x higher odds of positive COVID-19 tests on follow-up compared with stable indoor locations (adjustments for loss to follow-up, age, gender, race, 95% CI 1.86–20.77)	Low of note: weather conditions impacted outdoor group, incidents of residents going inside and unable to socially distance during storm. Limitations: high loss to follow-up, varying sample collection methods used due to test shortages at some points, wide confidence interval ranges. Study population of two urban shelters in same state—limited transferability.
Assessment of a Hotel-Based Protective Housing Program for Incidence of SARS-CoV-2 Infection and Management of Chronic Illness Among Persons Experiencing Homelessness (34) Huggett et al., 2021, United States	Retrospective cohort study April–September 2020	Varied homelessness (shelter, encampment, street), *n* = 259	Individual hotel rooms, healthcare workers available	C: homeless in shelters O: rates of COVID-19 infection	Retrospective analysis of people who were provided protective housing in individual hotel rooms. Participants were homeless who were deemed at risk of severe COVID-19 outcomes if they were infected (age, underlying health conditions). Healthcare workers on-site provided care, testing.	Homeless people in protective housing had lower risk of COVID-19 infection compared to shelter residents. 259 homeless people admitted to hotel, 201 included in protective housing cohort. 11 tested positive, 7 of these were within 5 days of admission. Overall incidence in hotel cohort was 54.7/1000 compared to 137.1/1000 among shelter residents in the same city (95% CI 125.1–149.1 per 1,000 people; *p* = 0.001). 11 of hotel cohort were transferred to hospitals for severe illness, no deaths. Improvements in chronic disease management, 51% housed after departure.	Low Estimation of non-intervention COVID-19 incidence may be inaccurate. Large portion of COVID-19 cases in hotel cohort were within 5 days of admission so possible overestimation of risk of infection in hotel. Selection bias risk high – sample recruited targeting their risk factors, voluntary. No unsheltered homeless who were approached to be recruited agreed to participate. Single site study. Some transferability to UK, implementation of hotel isolation. However, comparison is shelter rates of infection.
Of not passing: homelessness, addiction, mental health and care during COVID-19 Lenhard et al 2022 (35), UK	Qualitative study May 2020–April 2021	Across homeless support shelters *N* = 37 (30 service workers, 7 people experiencing homelessness)	Provision of accommodation to support homelessness, telemedicine to provide alternative access to healthcare during the pandemic	C: pre-pandemic O: experience of accommodation, COVID-19 on wellbeing and mental health	Semi-structured interviews conducted with both homeless service workers and member residents	Those suffering substance misuse and mental health issues found that as a result of the pandemic support was restricted. Digital options were not always suitable for those with challenging needs. One unexpected positive outcome was that some people had a better chance of securing more permanent housing as a result of having been provided housing at the start of the pandemic.	Moderate Though no mention of reflexivity or statement locating the researcher culturally, overall solid methodology, several quotes provided to support themes, qualitative methodology clearly explained and justified
COVID-19 among people experiencing homelessness in England: a modelling study (36) Lewer et al., 2020, England	Modelling study scenarios: 1st wave February–May 2020 2nd wave June 2020–January 2021	Temporary hostels, rough sleeping, night shelters, *n* = 46,565	Hotel accommodation (housing or isolation), reduced mixing with general population, infection control in settings, e.g., distancing, hand hygiene	C: no intervention, second wave scenarios O: rates of COVID-19 infection, hospitalisation, and mortality	Used a discrete-time Markov chain model, simulated under different scenarios varying the incidence of COVID-19 in the general population and use of prevention measures. First wave and future wave scenarios ran, each 200 times. Prevention measures including COVID-PROTECT (single room + bathroom) COVID-CARE (testing + medically supported accommodation for symptomatic individuals).	Prevention measures including hotel accommodation and medical care with COVID-19, reduced mixing with general population through lockdowns, and infection control strategies, successfully reduce adverse outcomes of COVID-19 in model. Model suggests 21,092 infections, 1,164 hospitalisations, 338 ICU admissions, and 266 deaths among homeless population prevented in the first wave. Even with no second wave in general population, if preventative measures are not continued, estimated additional 11,168 infections, 653 hospitalisations, 189 ICU admissions, and 165 deaths. If second wave but prevention measures continued, 1754 infections and 31 deaths estimated. If hotel accommodation and isolation rooms (PROTECT and CARE) only, rates are high but lower than without 3,654 infections and 54 deaths avoided	Moderate Uncertainty about COVID-19 rates and severity and homeless population, issues of modelling immunity, unknown actual size of homeless population, assumed no mixing between subgroups, assumed no changes in infectiousness. Based model on population of homeless from surveillance data in London only (rates, hospitalisation, mortality). While UK-based, numbers may not be fully representative of UK homeless populations.
“You Have a Place to Rest Your Head in Peace”: Use of Hotels for Adults Experiencing Homelessness During the COVID-19 Pandemic (37) Robinson et al., 2022, United State	Qualitative study March–May 2021	Two Hotels for those experiencing homelessness in New Haven *N* = 18	Hotels for those living in congregate shelter/unsheltered settings	C: Pre-pandemic O: account of people’s experiences of the hotels	Those living in shelters in New Haven were moved to single room ensuite hotel to contain transmission of COVID-19	On the whole participants stated that access to their own room and facilities (such as bathrooms) offered security, a greater sense of control, and empowered them to make positive changes for their health and wellbeing	Moderate Philosophical perspective unclear, no statement on reflexivity or one to local cultural and theoretical perspective of researchers, limited generalisability since only two hotels in one area focus of the study
Implementation of Baltimore City’s COVID-19 Isolation Hotel. Rosecrans et al. 2022, United States	Quasi-experimental study May 2020	Baltimore, isolation hotel for those experiencing homelessness *N* = 93 residents at peak of study	Isolation hotel for those experiencing homelessness-services for those suffering substance misuse	C: other isolation sites O: detection of COVID-19 among homeless population	Mode of recruitment to centre not clear, but 300 bed facility opened up to homeless in Baltimore, following collaboration between university of Maryland medical system and Lord Baltimore Hotel and Baltimore City Health Department	78% of residents did full isolation and quarantine routine, and just 6% of residents required transfer to hospital or higher intensity care-projections suggest hotel responsible for prevention of thousands of cases of COVID-19	Low Little outcome data, follow-up unclear, hard to determine who measured outcome data, no control group method of recruitment of participants also unclear or how demographic information on participants was obtained
Lessons Learned through Implementing SARS-CoV-2 Testing and Isolation for People Experiencing Homelessness in Congregate Shelters (38) Scott et al 2022, United States	Quasi experimental March to May 2020	Congregate shelters *N* = 52	COVID-19 testing	C: general population O: COVID-19 positivity rates among those experiencing homelessness	Community partners came together for the create and carry out a pilot testing alongside isolation in a homeless shelter, in order to review the viability of adopting such testing, in other homelessness facilities.	14 out of 52 residents tested positive, 13 residents with positive tests were moved to isolation hotels, 9 out of 13 were moved with 72 h of the test having been conducted.	Low Pilot study so small n, follow up unclear, no control group, comparison with general population not clear sample of the general population did not get COVID-19 testing in the same way
Comparing the initial Everyone In COVID-19 London response to the resurgence of Dec 2020–Feb 2021 (39) Story and Hayward, 2021, England	Observational report April 2020–February 2021	London-based homeless in hostels or hotel accommodation	Hotel accommodation, specifically *Everyone In*	C: between subgroups hostels and hotels O: rates of COVID-19	Limited methodology: Report on rates of COVID-19 collected in London homeless, some in hostels, and some in hotels connected to the everyone in initiative.	Hotel accommodation had a lower risk of COVID-19 infection than hostels for homeless population. Rise in cases in those living in hostel accommodation compared to emergency hotel accommodation and no fixed abode. Hostel group 5.6x increased risk of positive COVID-19 test compared to hotel accommodation. Likely connected to reduced capacity of hotels due to ending of service, leading to crowding of hostels which had worse infection measures	Low Absence/inaccuracy of available data on the size and characteristics of the accommodation and support offered to this population. Transferability n/a, UK based

### 2.5 Quality assessment

The internal validity of included studies was assessed by a single reviewer (FB or UO) using an appropriate critical appraisal tool based on study design (40–43). External validity was assessed to determine the transferability of results. For the overall assessment of the strength of evidence, a combined judgement of the designs, validity, and limitations of studies was applied (25).

### 2.6 Synthesis

Narrative synthesis (44) was performed, identifying types of interventions and their effectiveness. Due to the heterogeneity of the evidence in terms of study design, population of interest, interventions, and outcomes, meta-analysis was not possible.

Analysis of subgroups was intended if studies focussed on or specified between particular subtypes of people experiencing homelessness.

## 3 Results

### 3.1 Selection and overview of included studies

There were 8,233 initial hits from the search, and 4,183 references remained after deduplication. In total, 181 studies published before 2020 were removed before screening titles and abstracts. Full-text analysis was conducted for 230 articles. Five references identified through other sources were also screened.

**Figure 1:**
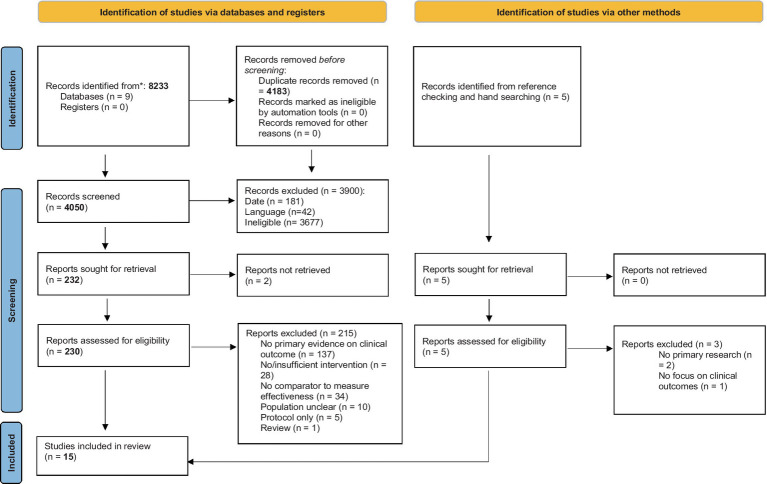
PRISMA flow diagram illustrating the process of selection of included studies (15).

Fifteen studies were included (Figure 1) (26–39, 45). There were six observational studies (27, 30, 31, 33, 34, 39), four pre−/post-intervention studies (26, 32, 38, 45), two qualitative studies (35, 37), and three modelling studies (27, 28, 34). Eleven of the included studies were from the US (27–34, 37, 38, 45) and three from the UK (35, 36, 39).

Interventions for people experiencing homelessness included symptom screening, testing, accommodation provision for positive cases, contact tracing, and provision of accommodation regardless of COVID-19 infection status. Studies often combine interventions together, especially modelling studies, which are detailed in Section 4.6.

The overall strength of evidence was low based on critical appraisal, study design, and transferability. Table 2 contains details of the included studies. There were insufficient studies to enable sub-group analysis by different types of homelessness characteristics.

### 3.2 Symptom screening, testing, and isolation accommodation provision

Four low-quality studies from the US and one from Canada looked at mitigating interventions that combined symptom screening, testing, and provision of accommodation for positive cases: two quasi-experimental studies (26, 38), one observational study (27), and two modelling studies (28, 29).

The first quasi-experimental study piloted a wastewater COVID-19 detection scheme at a large men’s homeless facility in Toronto (26). As a result of the scheme, COVID-19 activity was picked up before residents presented with symptoms. This was reported to have served as an important tool for prompt screening and outbreak management. The second quasi-experimental study piloted the impact of a COVID-19 testing scheme on 52 residents of a homeless shelter in Colorado (38). The success of the programme, with 93% of cases being moved to isolation centres within 3 days of a positive test, prompted the formation of more widespread COVID-19 monitoring schemes in the area (38).

One observational study conducted across 10 US homeless shelters reported successful implementation of an intervention of testing and referral for isolation of positive cases (27). However, this study reported issues regarding acceptance and adherence to testing, with just under half of eligible residents participating in testing and a quarter of participants adhering to twice-weekly testing.

Two modelling studies found conflicting results on the effectiveness of symptom screening and subsequent isolation, but both models suggested that PCR testing (and subsequent isolation) would decrease rates of COVID-19 (see also Section 4.6) (28, 29).

### 3.3 Alternative care sites

An alternative care site is defined as a medical treatment facility located in a non-traditional setting during a public health crisis (46). For this review, the term ACS is used for interventions that provide isolation accommodation that involves healthcare provision for individuals with COVID-19. An observational and a pre−/post-intervention study, both from the US and of low quality, assessed this type of intervention (31, 32).

One assessed the safety of hotel-based care systems for people experiencing homelessness and looked at adherence to isolation measures (31). People experiencing homelessness and mild COVID-19 infections were referred from other settings (e.g., hospitals) if they were unable to isolate safely. In total, 955 guests resided in hotel-based care, of which 81% completed their isolation. Premature discontinuation was most strongly associated with unsheltered homelessness (aOR = 4.5, 95% CI 2.3–8.6). Other significant associations included being under 40 years old, female, and of black ethnicity. In this study, 346 patients from hospitals were successfully referred to a hotel with healthcare, and 4% were readmitted for worsening COVID-19.

Another study compared hospitalisation rates of people experiencing homelessness before and after the implementation of a COVID-19 recuperation unit (CRU) (32). This intervention was an isolation space with healthcare provision specifically for people experiencing homelessness and those with substance use disorders. Over the study period, 226 people were admitted to the unit, with a 28% reduction in hospitalisations compared with before the intervention (risk ratio 0.72, 95% CI 0.63–0.82).

### 3.4 Contact tracing

One study focussed on contact tracing in people experiencing homelessness (30) and two other studies contained a discussion on the identification of close contacts (26, 31).

A US study of moderate quality reported difficult contact tracing for people experiencing homelessness and with COVID-19 (30). The researchers adopted a person-centred approach which required follow-up of positive cases to identify contacts and suggest this location-based approach may be more effective for people experiencing homelessness. Close contacts of people experiencing homelessness were more often unreachable compared to the general population (45% compared with 0% of the general population) (30). However, when tracing was successful, a higher proportion of contacts of people experiencing homelessness completed COVID-19 testing (62% compared to 42.5% in the general population). People experiencing homelessness reported fewer contacts per positive case compared to the general population (0.3 and 4.7, respectively). This low rate among close contacts was also reported across the US elsewhere (27).

Another US study found that quarantining of close contacts rather than a positive case was strongly associated with premature discontinuation of quarantine (31).

### 3.5 Provision of accommodation regardless of COVID-19 infection status

Eight studies included an intervention with the provision of housing for people experiencing homelessness regardless of their infection status. This included two comparative cohort studies (32, 33), one quasi-experimental study (45), one observational study (39), two qualitative studies (35, 37), and two modelling studies (Section 4.6) (27, 34).

A retrospective cohort study in the US looked at the impact of providing housing for people experiencing homelessness at high risk of COVID-19 complications (due to age and underlying health conditions) regardless of COVID-19 infection at the time of intervention (34). Of the 201 included in the cohort, the overall incidence of COVID-19 infection was 54.7/1000 compared to 137.1/1000 among shelter residents in the same city. Approximately 4% were transferred to hospitals for severe illness, and there were no deaths. Additionally, the intervention improved guests’ chronic disease management, and 51% were housed after departure from the study accommodation.

A prospective cohort study from the US investigated the effectiveness of different strategies to reduce the population density of shelter residencies (32). Two homeless shelters adopted different strategies: One set up temporary tents in the car park, and the other moved residents to indoor spaces such as recreational centres and hotels. The residents who had moved to temporary outdoor tents had a higher risk of testing positive for COVID-19 on follow-up than people who had moved to alternative indoor sites (aOR = 6.21, 95% CI 1.86, 20.77).

A quasi-experimental study piloted a COVID-19 isolation hotel in Baltimore, which served 93 homeless residents at its peak (45). This hotel also provided services for people experiencing homelessness and who had substance misuse problems. Though study quality was low, with few outcome data, the authors project that the hotel prevented thousands of cases of COVID-19 through the vast majority of its residents completing a full quarantine period. Just 6% required to transfer to a hospital (45).

An observational study from the UK explored a surge in cases of COVID-19 in the London homeless population living in hostels compared to those housed in the *Everyone In* hotels in the second wave of the pandemic (39). Residents in hostels had a 5.6 times increased risk of a positive test compared to those in emergency hotels. This difference was interpreted to be partly due to the general surge in cases with a more infectious strain (variant B117) and also due to the discontinuation of some *Everyone In* hotels, which led to a rise of hostel residents where infection strategies were not as well implemented.

One qualitative study reviewed the impact of the pandemic on those experiencing homelessness in the UK (35), including the effects of providing accommodation and changes in access to healthcare during the pandemic. People with substance misuse and mental health issues had less access to support during COVID-19 restrictions. Conversely, one unexpected positive outcome was that some people experiencing homelessness had a better chance of securing more permanent accommodation through being offered accommodation (35).

Another qualitative study of 18 residents, who had previously lived in unsheltered housing, examined the impact of hotels designed to curtail the spread of COVID-19 among people experiencing homelessness in New Haven (37). Participants described an increased sense of security from having private bedrooms and bathrooms, which in turn empowered the residents to implement health-promoting behaviours (37).

### 3.6 Modelling studies of multiple interventions

Three modelling studies looked at multiple interventions, based on people experiencing homelessness in England, UK (36) and the US (28, 29).

One study used a model to predict the impact of preventive measures on COVID-19 rates of infection, hospitalisation, ICU admission, and mortality for an estimated 46,565 people experiencing homelessness in England (36). The preventive measures modelled were hotel accommodation for isolation or housing, reduced mixing with the general population (lockdown measures), and infection control in homeless settings such as hand hygiene and social distancing. The model suggested that preventive measures avoided 21,092 infections and 266 deaths in people experiencing homelessness during the first wave of the pandemic (36). Furthermore, it predicted that even if there was no second wave in the general population, discontinuation of preventive measures would lead to an estimated additional 11,168 infections and 165 deaths. In the model, the provision of hotel accommodation and isolation rooms alone still prevented some infections, hospitalisations, and deaths but was less effective than combining lockdown measures and infection control strategies.

A modelling study based on 2,258 homeless shelter residents in a US city looked at symptom screening, regular testing, alternative care sites (ACSs), and temporary housing (28). The model indicated that daily symptom screening and provision of an ACS for isolation for COVID-19 were associated with 37% fewer infections. It was estimated that symptom screening and ACS were associated with 46% lower healthcare costs compared to no intervention predictions. Implementing PCR testing every 2 weeks further decreased infections but increased costs. The provision of housing and fortnightly PCR testing was the most effective intervention to reduce rates of COVID-19 (compared to no intervention, symptom screening, testing, and ACS) but was found to be the most expensive.

Conversely, another modelling study, based on populations of homeless shelters across three US cities, found that daily symptom screening was a poor mitigating intervention for COVID-19 transmission (29). This was indicated even when general population COVID-19 incidence rates were low or when combined with isolation accommodation. It was estimated that PCR testing twice per week for all residents improved the probability of averting an outbreak in homeless settings. However, this model found that in high-density settings or when background rates of COVID-19 were high, even multiple strategies showed very little improvement in preventing an outbreak of COVID-19.

## 4 Discussion

### 4.1 Principal findings

This review identified various interventions used to try to mitigate the risks of COVID-19 in people experiencing homelessness. Interventions often involved identifying people who may potentially have COVID-19, so that isolation spaces, an alternative care site, or urgent (re-) housing may be provided. Conflicting evidence was found on the benefits of symptom screening alone (28, 29), and contact tracing was difficult in this population (30).

Alternative care sites were successfully implemented to care for infected individuals and reduced hospital admission rates (31, 32). Accommodation provision for people experiencing homelessness regardless of COVID-19 infection was found (or modelled) effective in preventing the spread of COVID-19 (28, 33, 34, 36, 37). Some evidence suggests that lockdown measures that reduced mixing among people experiencing homelessness and with the general population also limited the spread of COVID-19 (36).

Evidence from modelling studies suggests that the implementation of multiple interventions involving various combinations of alternative care sites, housing, infection control strategies in communal spaces, and national lockdowns was more effective than implementing single measures (28, 29, 36).

### 4.2 Context of other literature

The COVID-19 pandemic had devastating health, social, and financial impacts on people globally (47–49) and severe impacts on healthcare systems (50). However, there is some evidence that policy and services for people experiencing homelessness have received more concerted focus, funding, and efforts in collaboration than before the pandemic (51). The finding that testing and isolation accommodation were successful in reducing rates of COVID-19 infection in people experiencing homelessness is similar to findings for the general population and for other vulnerable groups such as people in prisons or care homes (52, 53) and has become widely accepted as an effective means of preventing transmission (54, 55).

The benefit of housing people experiencing homelessness to stop the spread of COVID-19 likely derives from providing individual spaces for people to isolate or spend lockdown and the ability to ensure adherence to infection control measures (56). Additionally, the stability and safety provided may have helped people’s background health, across physical, social, and mental health domains. However, people sleeping rough have strong feelings of marginalisation and mistrust of authority (57). The finding that rough sleepers were less likely to complete their isolation period (31) is consistent with findings in the UK of people who had come from rough sleeping not wishing to remain in hotel accommodation (56).

### 4.3 Strengths and limitations of the available evidence base

Overall, few studies met the eligibility criteria of this review. Many studies contained no or very limited primary research or comparison and were excluded. The included studies were mostly of low quality, with only two studies deemed moderate quality (30, 36), which limits the interpretation of findings. The studies included did not always specify enough detail on the population studied, and some had low adherence to the intervention being studied.

The modelling studies, of which there are three in this review, are hypothetical in nature, based on assumptions about COVID-19 (e.g., period of infectiousness) and factors relating to people experiencing homelessness (e.g., no mixing between subgroups) and did not account for the impact of uncertainty in these assumptions. However, the strength of these studies is the larger population size than in the other study designs included.

There was no evidence found on any interventions for people who are in precarious or unstable housing, often termed “hidden homelessness” (58).

### 4.4 Strengths and limitations of the review

This review has limitations in its methodology. Studies not published in English were excluded. Additionally, only 10% of potential studies were screened by a second reviewer, and full-text analysis and quality assessment were done by a single reviewer. A well-developed set of inclusion criteria and use of standardised critical appraisal tools were used to combat this limitation (40–43). In contrast to a developing literature base on the impacts of COVID-19 on people experiencing homelessness (7), there remains relatively little evaluation of interventions to mitigate these impacts, making the drawing of conclusions limited.

### 4.5 Implications for policy and practice

Although service collaboration and funding in the homelessness sector improved during the pandemic to reduce the risk of COVID-19 in people experiencing homelessness, there are now concerns that government funding for these interventions is decreasing. Furthermore, rates of homelessness are increasing due to increased costs of living (59). To prevent the risks of COVID-19 outcomes in people experiencing homelessness, as well as the health inequalities they experience, continuation of accommodation provision and healthcare is vital (28, 33, 34, 36, 37). There is, however, concern that interventions have been applied or advocated on a “one-size-fits-all” basis and are not sufficiently flexible or tailored to a wide range of individual circumstances and needs (35). Studies on interventions in shelters (mainly in the US) may initially appear to have less transferability to other settings, but there is the potential for learning and adapting. Effective interventions in congregate living settings may be transferable to homeless populations in settings such as hostels and hotels with multiple occupants.

### 4.6 Implications for future research

The relative paucity of research in this review indicates that robust research is required to evaluate the effectiveness of interventions in people experiencing homelessness during COVID-19 or other potential pandemics or public health crises. In the UK, there is very limited evidence on the true impact of *Everyone In*—a prominent policy for people experiencing homelessness. There are many official publications that report on the success of the *Everyone In* (20, 56, 60), but conclusions are almost entirely based on the modelling study by Lewer et al. (36). The *Everyone In* initiative is under-researched, especially since the mortality rates used in the model are based on a small sample of people experiencing homelessness early in the pandemic.

Research could be structured around risk scenarios—i.e., “baseline” when there is low incidence, “defend” when there are consistently rising levels of infection, and “outbreaks” in more localised or contained settings, as suggested, for example, in care home communities (61). Research should be also conducted reviewing the prevalence of long-term impacts of COVID-19 on people experiencing homelessness such as long COVID and mental wellbeing and interventions to mitigate these outcomes (62, 63).

## 5 Conclusion

This systematic review summarises the evidence on interventions for people experiencing homelessness and their effectiveness in mitigating the impacts of COVID-19 and its outcomes. Common strategies included combining identification of potentially positive cases with isolation accommodation and provision of individual housing. Interventions appeared to decrease the transmission of COVID-19 and reduce the burden on hospitals. The evidence base in this review must be interpreted with caution due to the low volume of eligible studies and the low quality of evidence within the review. From the evidence available, the provision of isolation accommodation and housing of individuals not in shelters should be continued. However, it is essential for this population that further research is conducted to help guide policy and practice in the management of the ongoing COVID-19 pandemic and potential future pandemics.

## Author contributions

OO: Formal analysis, Investigation, Methodology, Writing – original draft, Writing – review & editing. FB: Formal analysis, Investigation, Methodology, Writing – original draft. BS: Formal analysis, Methodology, Writing – original draft. DW: Conceptualization, Investigation, Methodology, Project administration, Supervision, Validation, Writing – original draft, Writing – review & editing. RL: Conceptualization, Supervision, Writing – review & editing. AE: Conceptualization, Formal analysis, Funding acquisition, Investigation, Methodology, Resources, Supervision, Validation, Writing – original draft, Writing – review & editing.

## Appendix

Search strategy of databases.

Medline via OVID 18.11.22.

**Table:**

	String line	Number of results
1	exp Coronavirus/	160,423
2	COVID-19/	210,509
3	((corona* or corono*) adj1 (virus* or viral* or virinae*)).ti,ab,kw	5,338
4	(coronavirus* or coronovirus* or coronaviri* or 2019-nCoV or 2019nCoV or nCoV2019 or nCoV-2019 or covid-19* or covid19* or ncov* or n-cov* or HCoV* or SARS-CoV-2 or SARSCoV-2 or SARSCov2 or SARS-CoV2 or severe acute respiratory syndrome).ti,ab,kw	341,577
5	((outbreak* or pandemic* or epidemic*) adj10 (wuhan or hubei or china or Chinese or Huanan)).ti,ab,kw	11,698
6	1 or 2 or 3 or 4 or 5	359,003
7	exp homeless persons/ or exp. homeless man/ or exp. homeless youth/ or exp. homeless woman	10,711
8	(homeless* or unhouse* or unshelter* or roofless* or houseless* or “sleeping on the street*” or “living on the street*” or “sleeping rough” or “living rough” or “rough sleep*” or “street person*” or “street people” or “street liv*” or “without a roof”).ti,ab.	13,499
9	(“no fixed address*” or “no fixed abode” or “unstable hous*” or “hous* instability” or “lack of hous*” or “vulnerably hous*” or “night shelter” or “transition hous*” or “supported hous*” or “emergency hous*” or “emergency shelter*” or “temporary accommodation” or “emergency accommodation” or “insecure accommodation” or “precarious hous*” or “seeking shelter” or “couch surf*” or “sofa surf*”).ti,ab	2,225
10	(street dwell* or improvised dwell* or shelter dwell* or sleeping out* or street involved).ti,ab	301
11	((without or no or “lack of” or inadequate*) adj1 (hous* or accommodation* or shelter* or hostel* or dwell*)).ti,ab	1819
12	((homeless* or street or transient* or marginal* or vulnerabl* or temporary or unstabl* or vulnerabl* or insecure or support* or transition*) adj2 (hous* or accomondation* or shelter* or hostel* or dwell*)).ti,ab.	5,687
13	7 or 8 or 9 or 10 or 11 or 12	21,709
14	6 and 13	949
